# Tincture of Calabar Bean

**Published:** 1873-09-01

**Authors:** 


### TINCTURE OF CALABAR BEAN.

Editors of Examiner:

To answer many inquiries as to the strength and method of preparing the tinct. of calabar bean, to which reference was made by me in a recent article upon meningitis, I beg you to insert the following extract from *The Pharmacist*, of Sept., 1872, kindly furnished by Mr. A. E. Ebert, chemist, corner 12th and State streets, Chicago, from whom a reliable article can always be had.

Respectfully yours,
J. H. HOLLISTER.

"Take of—

℞.—Calabar bean, in very fine powder, ℥ xvi.

Acetic acid, ℥ ss.

Strong alcohol.

Water of each, 9 O.

Mix the acid with three pints of water, pour this upon the powder, and set the mixture aside for a few days in a warm place; then add to it three pints of strong alcohol (95 per ct.) and macerate it for a few days longer. After this pour part of the mixture upon a muslin strainer; press the liquid out, and pack the residue of the strainer firmly into a cylindrical glass percolator, having a broad base; pour on to this foundation the rest of the mixture, together with the strained liquid, and then a mixture of equal parts of strong alcohol and water, until spirits percolate.

"This tincture conforms in the proportion of two troy ounces to one pint of the finished product, making it similar in this respect to the strength of most of the other tinctures."

